# Visualising the strength development of FICP-treated sand using impedance spectroscopy

**DOI:** 10.1038/s41598-024-73938-z

**Published:** 2024-10-15

**Authors:** Jamal Ahmad, Mohammad Arsalan Khan, Shakeel Ahmad, Meshel Q. Alkahtani, Mohammad Mursaleen, Saiful Islam

**Affiliations:** 1 Department of Civil Engineering, Zakir Husain College of Engineering and Technology, Aligarh Muslim University, Aligarh, 202001 India; 2 Geomechanics and Geotechnics Group, Kiel University, 24118 Kiel, Germany; 3 New Era and Development in Civil Engineering Research Group, Scientific Research Center, Al-Ayen University, Thi-Qar, Nasiriyah, 64001 Iraq; 4 Civil Engineering Department, College of Engineering, King Khalid University, 61421 Abha, Saudi Arabia; 5 China Medical University Hospital, China Medical University (Taiwan), Taichung, 40402 Taiwan

**Keywords:** FICP process, AD5933 chip EMI technique, Sand, Piezoelectric patch, Statistical metrics, Civil engineering, Optical spectroscopy

## Abstract

Fungal Induced Calcium Carbonate Precipitation (FICP) is a novel method used in geotechnical engineering that enhances the engineering properties of sand by using the potential of fungal activity. This research is the first attempt to monitor the strength of FICP treated sand using embedded Piezoelectric (PZT) patch based Electromechanical Impedance (EMI) spectroscopy. In the past, the strength of such treated sand has been determined through the destructive methods like Unconfined Compressive Strength (UCS) test. In this study, the sand is mixed with the filamentous fungus *Aspergillus Niger* and the cementation solution (urea and $\:{\text{C}\text{a}\text{C}\text{l}}_{2}$ in the ratio of 1:1) is injected after every 24 h. Results recorded from the cost-effective EVAL AD5933 chip indicate that the shifting of frequency impedance signals in each phase is in good alignment with UCS and calcium carbonate content (CCC). Following the 28-day treatment period, the treated sand achieves a maximum UCS of 3.93 MPa, accompanied by a CCC of 15.19%. In order to correlate EMI signals with treatment cycles, UCS, and CCC, various multi linear regression (MLR) equations for statistical metrics like root mean square deviation (RMSD), mean absolute percentage deviation (MAPD), and correlation coefficient deviation (CCD) are developed. Additionally, Scanning Electron Microscopy (SEM) and Energy Dispersive X-ray (EDX) analyses have been conducted to observe the success of the FICP process in the sand.

## Introduction

Soil performs an essential part in modern construction, serving as the base of civil infrastructures (e.g., high rise structures, highways, bridges, etc.). However, the bearing capacity of soils particularly poorly graded sand can be unsatisfactory for construction uses. Traditionally, soil can be improved by mechanical methods (e.g., compaction, pre-loading, vibration) or through the chemical and synthetic additives (e.g., cement, lime, fly ash, industrial wastes, organic compounds, geosynthetics)^1^. Regardless their established effectiveness, these traditional approaches are now being constantly examined because of their substantial cost and energy demand along with their negative impacts on the ecosystem^2^. Researchers are currently focussing on biomediated approaches like microbial induced calcium carbonate precipitation (MICP), which is entirely different from conventional techniques. MICP is a promising, economical, sustainable solution in comparison with the current soil strengthening approaches^3^.

MICP is usually performed by specific microbes like bacteria (Bacterial induced calcium carbonate precipitation) or fungi (Fungal induced calcium carbonate precipitation). Fungal induced calcium carbonate precipitation (FICP) may offer many calcium carbonate nucleation sites because it has a filamentous shape compared to bacteria, which means that it can be highly effective at biomineral formation^4,5^. Fungi can survive in hard conditions and get nutrients from the air and rain. They can also grow on rocks, cement, and mortar^6^. The main metabolisms that occur in MICP are urea hydrolysis, ammonification, denitrification, sulfate reduction and photosynthesis. Within these methods of metabolism, urea hydrolysis is simple, and demonstrated higher calcium carbonate content (CCC) about 20–80% in contrast with other processes of metabolism^7^. The method of MICP via urea hydrolysis is that urea can be decomposed into carbon dioxide and ammonia by urease enzyme secreted from the microbe’s cells. In the urea hydrolysis process, microbes are able to use urea as their energy and nitrogen source for respiration, during which carbon dioxide is also created. In alkaline environment resulting from ammonia, carbon dioxide is transformed into carbonate ions. At the same time, microbes possess the ability to take calcium ions from the surrounding environment onto the surface of their cells with negative charge. Following that, once calcium ions meets carbonate ions, enormous amounts of calcium carbonate crystals occur and accumulate on the surface of the microbial cells, which leads to cementation of sand grains^2,8^.

1 $$\:{{\text{C}\text{O}(\text{N}\text{H}}_{2})}_{2}+{\:\text{H}}_{2}\text{O}\:\underrightarrow{\text{U}\text{r}\text{e}\text{a}\text{s}\text{e}\:\text{p}\text{r}\text{o}\text{d}\text{u}\text{c}\text{i}\text{n}\text{g}\:\text{m}\text{i}\text{c}\text{r}\text{o}\text{b}\text{e}}\:{\text{N}\text{H}}_{3}\:+\:{\text{H}}_{2}\text{N}\text{C}\text{O}\text{O}\text{H}\:$$

2 $$\:{\text{H}}_{2}\text{N}\text{C}\text{O}\text{O}\text{H}\:\to\:{\:\text{N}\text{H}}_{3}\:+\:{\text{C}\text{O}}_{2}$$

3 $$\:{\text{N}\text{H}}_{3}\:+\:{\text{H}}_{2}\text{O}\:\to\:\:{\text{N}\text{H}}_{4}^{+}\:+\:{\text{O}\text{H}}^{-}$$

4 $$\:{\text{C}\text{O}}_{2}\:+\:{\text{H}}_{2}\text{O}\:\to\:\:{{\text{H}}_{2}\text{C}\text{O}}_{3}\:\to\:\:{\text{H}}^{+}\:+\:{\text{H}\text{C}\text{O}}_{3}^{-}\:\to\:\:{2\text{H}}^{+}\:+\:{\text{C}\text{O}}_{3}^{2-}$$

5 $$\:{\text{C}\text{a}}^{2+}\:+\:{\text{C}\text{O}}_{3}^{2-}\:\to\:\:{\text{C}\text{a}\text{C}\text{O}}_{3}$$

FICP is mainly influenced by the following key factors, include the species of fungus, concentrations of fungal and cementation solutions, pH, temperature, sand properties, and treatment cycles^9^. Monitoring the FICP treatment process constantly is important to assessing its effectiveness and assuring desirable modifications to sand properties, making it valuable for predicting geohazards like landslides and following suitable mitigation measures. In the past, unconfined compressive strength (UCS) tests have been used to study the strength development of FICP-treated sand, however these are destructive and give data only at certain points in a period of time, require extensive manpower and specialized tools^10^. This study suggests a novel way using structural health monitoring (SHM) by PZT-based electromechanical impedance (EMI) spectroscopy, which offers several advantages over traditional approaches^11–13^. EMI spectroscopy, employing an EVAL AD5933 chip and a PZT patch that works as both actuator and sensor, offers a non-destructive method for monitoring the FICP process. Any alteration in the mechanical properties in a sand produces variations in the mechanical impedance, which also induces changes in the electrical admittance of the PZT patch embedded into the sand. Through monitoring variations in the EMI signal during treatment, a deeper understanding of the increase in strength can be achieved. The variations from anticipated EMI patterns can suggest possible problems with the FICP process, which permits prompt remedial action. The AD5933 impedance chip’s wide availability and affordability made this EMI method feasible in comparison to UCS testing.

Current literature reveals a critical knowledge gap regarding the integration of the FICP treated sand’s strength monitoring with EMI technique. However, EMI has already been utilized as a non-destructive testing technique to evaluate the mechanical characteristics of soils, providing valuable insights into parameters such as strength, stiffness, density, freeze thaw and moisture content^14–18^. Zhang^14^ studied the freeze-thaw process and strength with PZT sensors gives an integrated approach for soil compressive strength assessment. The horizontal and axial variations of resonant peaks, along with the application of statistical metrics like RMSD, MAPS, and CCD, gave a thorough quantitative assessment of soil compressive strength during the freeze-thaw process. Lan^15^ investigated spherical smart aggregates (SSAs) presented as a novel solution for real-time monitoring of soil water content. This method not only extended the variety of readily accessible sensors but additionally gave an option for improved precision in soil water content assessments. This study showed the broad application potential of PZT transducers in different soil monitoring situations. Wu^16^ worked on Compactness Measuring Sheet (CMS) embedded in soil for compression tests provides helpful insights into soil compaction monitoring. The observation of reduced peak magnitudes and specific frequency changes in conductance signatures throughout compression tests gives a tangible signal of increased soil compaction. The use of statistical metrics such as RMSD, MAPD, and CCD, further improved the study’s reliability, offering quantifiable indicators for changes in conductance signatures. Wu^17^ adopted lead PZT transducers as the EMI technique gives a robust basis for soil monitoring. The application of waterproof-insulated PZT transducers embedded in both cohesive and sandy soils displays adaptability. The noted rightward movement of peak frequency and decreasing peak magnitude when water evaporates presents tangible proof of the ability of the EMI technique to detect variations in soil moisture content. The addition of statistical metrics such as RMSD and MAPD improves the investigation through analyzing changes and at the same time confirming the accuracy of PZT transducer-based monitoring.

For EMI monitoring, Impedance analyzers have been used in research over the past two decades to monitor the strength of various materials, particularly concrete and soil. However, their high cost (USD 20,000 to 40,000) and lack of portability make them unsuitable for real-world SHM applications^19^. This study utilizes the EVAL AD5933 chip, which is cheap (US$ 160), lightweight, and compact. The comparison of specifications between LCR meter/impedance analyser and EVAL AD5933 chip is presented in Table 1. Several researchers have already demonstrated AD5933 chip potential through various applications^20–23^. To the best of the authors’ knowledge, this is the first study that integrates impedance spectroscopy with FICP treatment of sand. Our study aims to investigate the effectiveness of FICP as a soil-strengthening technique and evaluate the feasibility of embedded PZT patches along with the AD5933 impedance chip for continuous monitoring of the whole process. Through examining the effect of different time periods (7, 14, 21, and 28 days) on UCS and CCC inside the treated sand, we aim to find the critical periods over that calcium carbonate precipitation gets the most significant. The AD5933 impedance chip works in a frequency that ranges from 30 to 100 kHz, which allows a thorough investigation of the EMI signal variations over the course of time. The research involved stands distinct for a number of factors. Firstly, it is to integrate FICP with EMI spectroscopy, offering a non-destructive and continuous monitoring method that greatly improves limitations of traditional methods. Second, we adopt Aspergillus Niger filamentous fungus in the MICP process, a less widely used microbe in these types of applications. This option possibly opens up the path for more environmentally friendly and economical FICP treatments. Furthermore, we ensure high precision of the EMI signatures by means of an extensive calibration process via EVAL-AD5933 evaluation board and a commercial LCR meter (LR-6200). This level of authentication by calibration is important to verify the accuracy of EMI-based monitoring. In order to relate EMI signals with treatment cycles, UCS, and CCC, we establish different multi-linear regression (MLR) equations for statistical metrics such as RMSD, MAPD, and CCD. Moreover, we confirm the success of the FICP treatment through Scanning Electron Microscopy (SEM) and Energy Dispersive X-ray (EDX) analyses, giving an in-depth analysis of the microstructural alterations in the treated sand.

**Table 1:** Comparison of technical specifications of LCR meter/Impedance analyzer and EVAL AD5933 chip.

Specifications	LCR meter/Impedance analyzer	EVAL AD5933
General	Professional precise laboratory measurement equipment Calibrated and ready to use	The AD5933 is a high precision impedance converter system solution that combines an on-board frequency generator with a 12-bit, 1 MSPS, analog-to-digital converter (ADC). Needs calibration before use
Frequency ranges	4 Hz to 5 MHz (5 digits setting resolution, minimum resolution 10 mHz)	Up to 100 kHz
Measured parameters	Z, Y, θ, Rs (ESR), Rp, Rdc (DC resistance), X, G, B, Cs, Cp, Ls, Lp, D (tanδ), Q	Z as real and imaginary parts, other parameters need to be calculated
Measurement range (@Z)	100 mΩ to 100 MΩ, 12 ranges (All parameters are determined according to Z)	1 kΩ to 10 MΩ
Accuracy (@Z)	± 0.08%	± 0.5%
Power supply	90–264 VAC, 50/60 Hz, 150 VA max.	2.7 V to 5.5 V DC, *<* 1 W
Dimensions and mass	330 mm × 119 mm × 307 mm, 5.8 kg	@Chip – ~ 6 mm × 8 mm for 16-lead SSOP SMD package, few grams @Evaluation board – ~ 80 mm × 80 mm, 237 g
Cost	20,000–40,000 USD	160 USD

## Materials and methods

### FICP treatment of sand

#### Fungal strain selection and cementation solution

The experiment utilized fungal strain *Aspergillus niger* fungus, obtained from the Microbial Type Culture Collection and Gene Bank (MTCC) in Chandigarh, India. This strain was maintained on potato dextrose agar (PDA) slants stored at a cool temperature of 4 °C. To prepare a spore suspension, 10 ml of sterile distilled water was added to the surface of the PDA slant and gently scraped with a sterile loop. This suspension was then transferred to a sterile 500 ml flask containing 200 ml of sterile MGYP medium, which is composed of malt extract, glucose, yeast extract, and peptone. The culture was incubated at 25 °C on a rotary shaker at 200 rpm for 96 h to allow for growth. The concentration of the cementation solution plays a crucial role in the FICP process. Typically, concentrations ranging from 0.25 to 2.5 M are used. Higher concentrations generally lead to increased precipitation of calcium carbonate, which strengthens the sand. However, exceeding a concentration of 1.0 M can have a retarding effect, actually decreasing the amount of calcium carbonate precipitation^24^. In this study, a cementation solution composed of a 1:1 ratio of urea and CaCl_2_ in deionized water was employed.

#### Sand properties

**Fig. 1:**
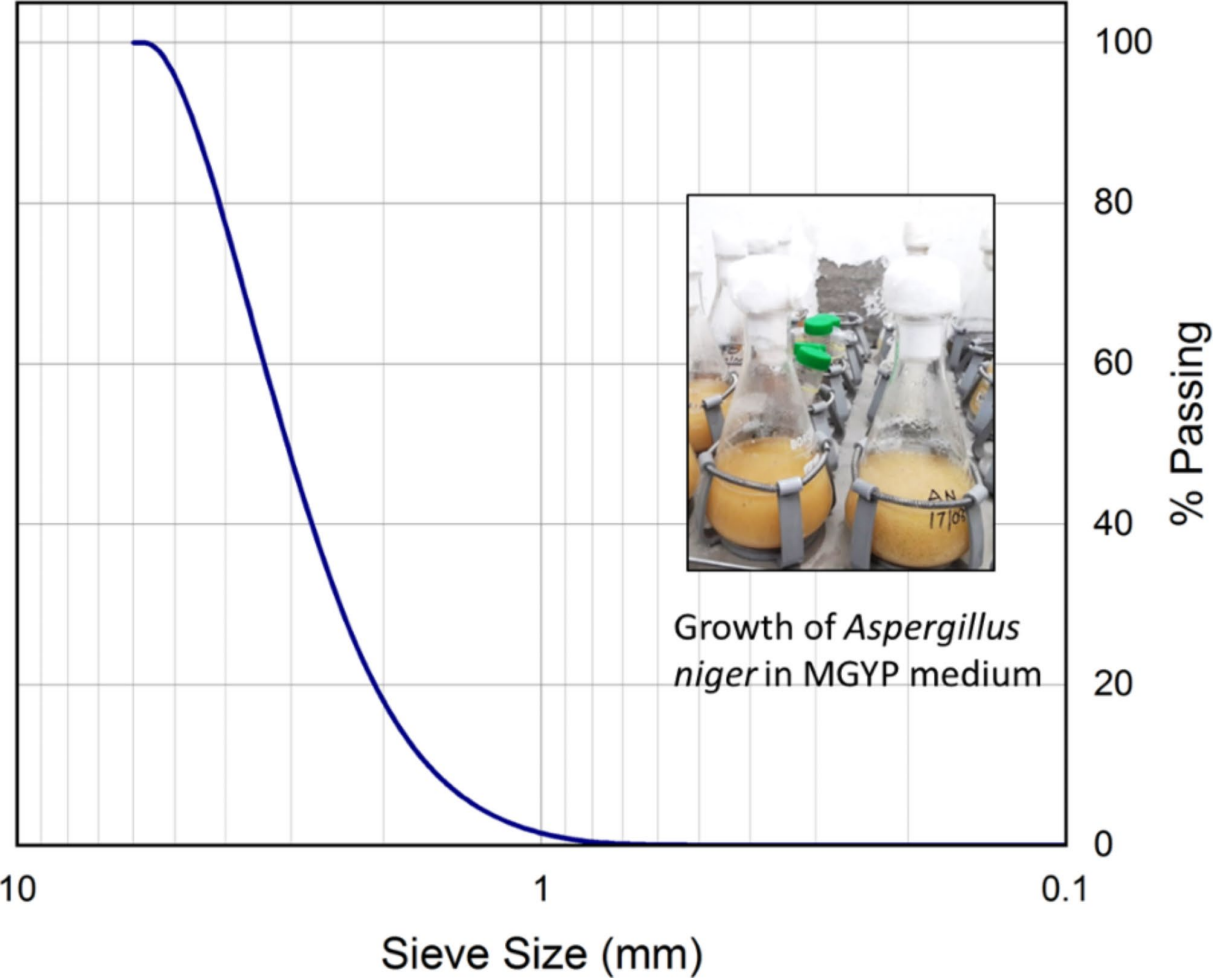
Particle size distribution curve of sand used in this study.

The study employed locally-sourced coarse pit sand, commonly known as Badarpur, a material widely used within India’s construction sector. To eliminate oversized particles and debris, the sand was first passed through a 4.75 mm sieve. It was then oven-dried at 105 °C for 24 h to ensure complete removal of moisture. Sieve analysis determined the sand’s particle size distribution (Fig. 1). Based on the Unified Soil Classification System (ASTM D2487)^25^, the sand was categorized as poorly graded coarse-grained sand (SP). Further analysis revealed a coefficient of uniformity (C_u_) of 1.907 and a coefficient of curvature (C_c_) of 1.15.

#### FICP treatment procedure

**Fig. 2:**
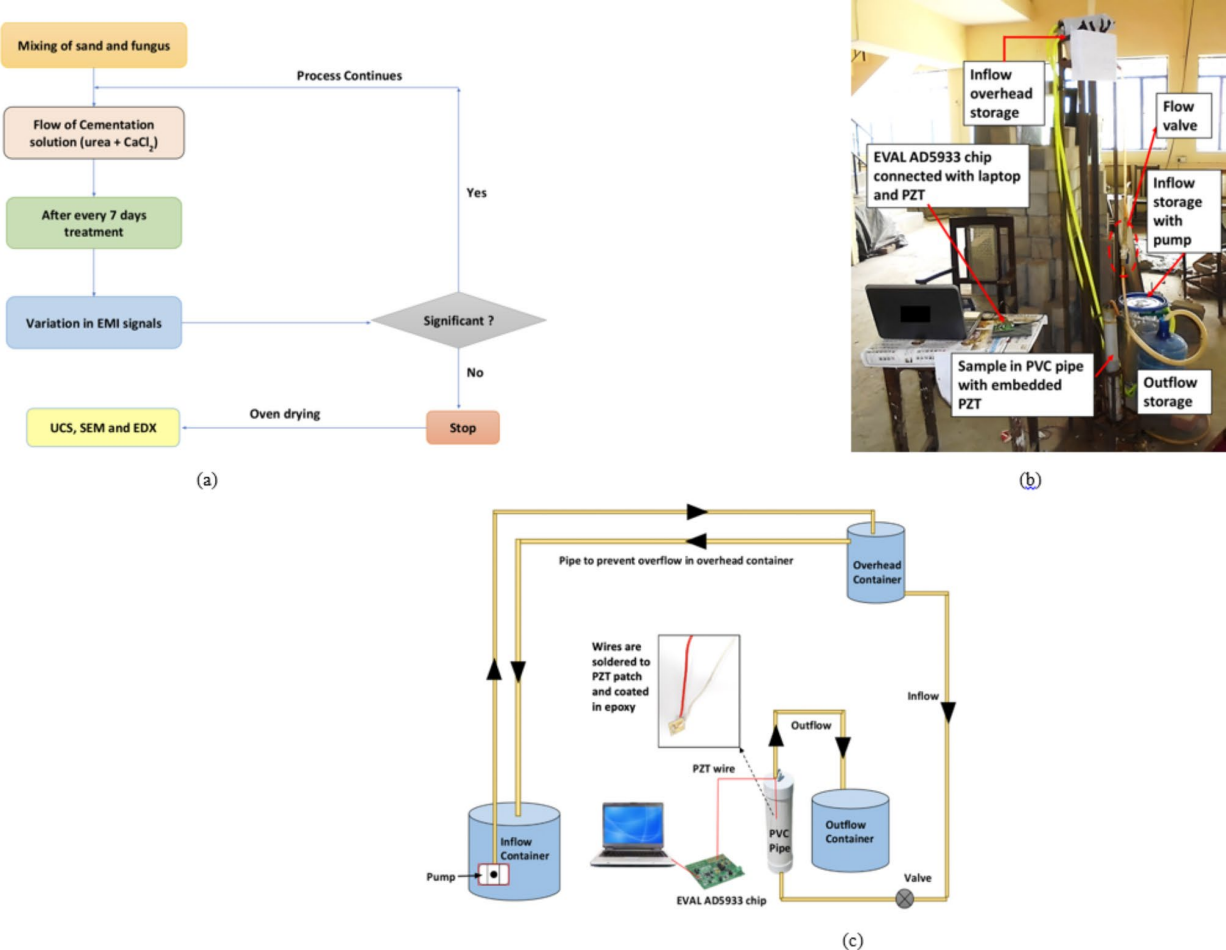
(**a**) Flow chart of treatment and data acquisition process, (**b**) Typical experimental setup, (**c**) Schematic diagram of experimental procedure.

A cylindrical mould (diameter: 38 mm, height: 500 mm) made of PVC was used to hold and shape the sand specimens during the experiment (Fig. 2). Three small tubes linked the top and bottom of the mould to enable the injection and drainage of a cementation solution for treatment. To avoid clogs and maintain continuous movement, both end caps were lined with fine mesh and filter paper. In addition, the inside of the mould was greased for simple extraction of the treated sand samples after the procedure was completed. The experiment started with the preparation of loose, coarse sand. The sand was completely dried by heating it in an oven at 105 °C for 24 h to remove any moisture. It was then carefully mixed with a fungal solution to ensure even distribution. During the pouring of the sand, a PZT patch was carefully placed 100 mm from the top of the PVC mould. This location ensured that the PZT patch was embedded within the sand matrix to record appropriate EMI data throughout the experiment. This sand was subsequently left undisturbed for 2 h, allowing the sand and fungal solution to interact. Following that, a cementation solution having equal amounts (1 M) of urea and calcium chloride was slowly added from the bottom of the cylinder. This upward movement guaranteed the expulsion of trapped air and even distribution of the solution. The solution moved through the cylinder in a constant hydraulic head, provided constantly from a tank 2.5 m above. The procedure of injection was carried out every 24 h. To promote and maintain the growth of fungal biomass within the sand, 200 ml of MGYP growth media was injected after every 7 days. Samples were regularly extracted for monitoring purposes (days 7, 14, 21, and 28). After the treatment, the sand sample was carefully taken out from the cylinder, cut into smaller cylinders (diameter: 38 mm, height: 76 mm), and rinsed with distilled water to eliminate any extra calcium carbonate or residual chemicals. At last, the samples were dried fully in an oven at 105 °C and used for UCS testing and CCC analysis.

### Impedance measurement setup

#### PZT type

In the present research, a 10 × 10 × 0.2 mm PZT-5 H patch was selected as the piezoelectric element. PZT-5 H is an exceptionally well lead zirconate titanate (PZT) ceramic recognised for its remarkable piezoelectric characteristics (Table 2). These features make highly sensitive and precise EMI signal extraction, necessary for observing the strength increase in MICP-treated sand. The resonant frequency of PZT-5 H has been assessed for compatibility with the proposed impedance spectroscopy setup, offering a balance among sensitivity and operational stability. A good conductive wire was selected for optimal electrical and mechanical performance, and then carefully soldered to the PZT patch to ensure an effective, stable connection. A thin, even coating of adhesive was next applied to the PZT patch for safety from damage and short-circuiting and rigidity. The patch and adhesive had been dried for a period of three days at ambient temperature.

**Table 2:** Properties of PZT used in this study for electromechanical impedance monitoring.

Properties	PZT type	Relative dielectric constant	Piezoelectric “d” coefficients relate the strain produced/electric field applied		Coupling coefficient		Density	Elastic (Young’s) modulus
Notations		$\:{K}_{3}^{T}$	*d*_*33*_	*d*_*31*_	*k*_*33*_	*k*_*31*_	$\:\delta\:$	$\:{Y}_{3}^{E}$
Values	5 H	3800	650 × 10 ^−12^	-320 × 10 ^−12^	0.75	0.44	7800	5.0 × 10 ^10^

#### EVAL -AD5933 chip and calibration

**Fig. 3:**
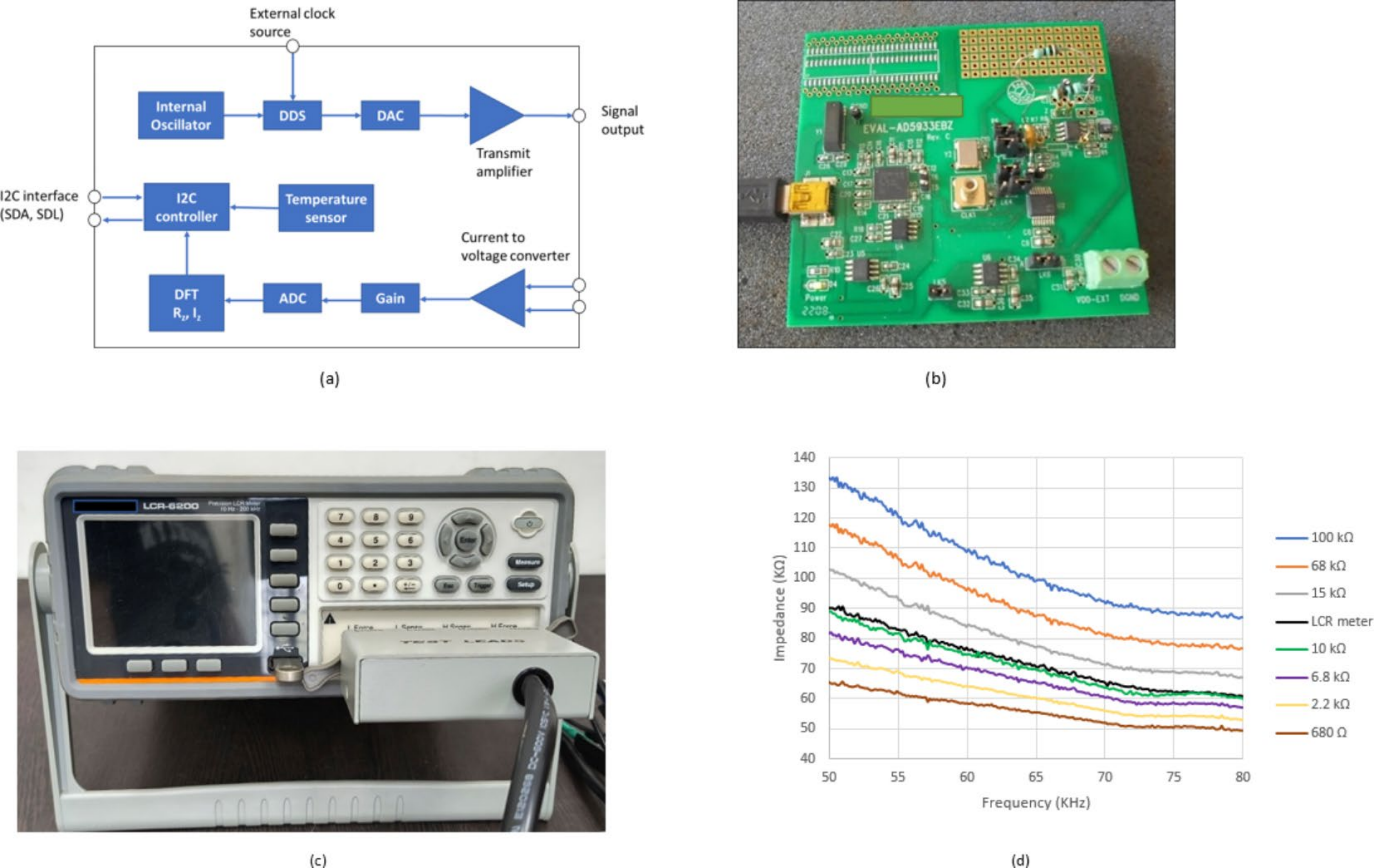
(**a**) Block diagram of AD5933 chip, (**b**) EVAL AD5933 chip, (**c**) LCR meter (**d**) Calibration of AD5933 with different resistors and LCR meter (LCR-6200).

The AD5933 is an integrated circuit from Analog Devices that functions as an impedance analyzer for a limited frequency range, typically from a few Hertz to 100 kHz. This chip incorporates several key components: an internal oscillator, a signal generator (DDS), converters for digital to analog (DAC) and analog to digital (ADC), amplifiers for transmission and current-to-voltage conversion, a gain control block, a digital signal processing engine with discrete Fourier transform (DFT) capability, and an I2C bus controller (Fig. 3a). In operation, the DDS and DAC work together to generate an analog sweep of frequencies. This sweep is then amplified and applied as the excitation signal to the impedance under test. The current response signal, along with current from a gain resistor (R_FB_), is converted to a voltage by the current-to-voltage converter. This voltage is then amplified and processed by the ADC and DFT engine. The DFT engine extracts the real (R_Z_) and imaginary (I_Z_) components of the impedance. The AD5933 communicates with a microcontroller via the I2C serial data bus. The real and imaginary impedance data from the DFT engine are transmitted to the I2C controller, which can then forward this data to the microcontroller for further processing. The I2C bus is also used to configure the AD5933’s operating parameters (such as frequencies and gain) and to read temperature data from an internal sensor. An evaluation board is available that allows the AD5933 to connect to a PC via USB. This board includes additional circuitry to facilitate communication. The evaluation board comes with dedicated software developed by Analog Devices. This software enables users to set operating parameters, perform calibration, conduct measurements, and store the measurement data. One important aspect to consider is that the AD5933 (device shown in Fig. 3b) requires calibration before measurements can be made. This calibration step is considered a drawback of this measurement approach, as it adds complexity to the process.

Previous research investigated the effectiveness of resistor-based calibration for measuring the electrical impedance of piezoelectric transducers bonded to structures^26^. The findings, indicated that the AD5933 chip could be calibrated using a resistor for impedance measurements within a limited frequency range. However, the calibration resistor value needs to be carefully chosen based on the specific piezoelectric transducer’s impedance. In this study a professional laboratory LCR meter (LCR-6200 by GWINSTEK) alongside the AD5933 chip to measure the impedance of a piezoelectric transducer. Calibration was performed using resistors of equal value (R_CAL_ and R_FB_) ranging from 680 Ω to 100 kΩ. The results, presented in Fig. 3c, compared impedance measurements obtained with the LCR meter and the AD5933. The best agreement between the LCR meter and AD5933 measurements was achieved with a 10 kΩ calibration resistor (which was also the value used for R_FB_). According to the Eq. (6) calibrating resistance should be equal to ~ 10.6 kΩ, so the formula can be also used for the piezoelectric transducer. The piezoelectric transducer’s impedance magnitude (|Z|) ranged between 60 kΩ and 88 kΩ. Interestingly, the results revealed that excessively low or high calibration resistor values resulted in significant errors, particularly affecting the identification of resonant peaks at both low and high frequencies. These findings demonstrate that calibration with an appropriately chosen resistor allows the AD5933 to measure impedance spectra with a high degree of similarity to those obtained with the LCR meter, in terms of shape, trend, and impedance values. It is important to note that even with optimal calibration, the measured impedance values from the AD5933 might not perfectly match those from the LCR meter. Potentially, even better agreement could be achieved by finding the absolute optimal calibration resistor value or even using a potentiometer. However, in the context of structural health monitoring (SHM) using the electromechanical impedance method, the focus is not on achieving perfect accuracy but rather on ensuring repeatability in the measured impedance spectra. The crucial aspect is to clearly identify the resonant peaks in the impedance characteristics and ensure their frequencies align with the actual values^27,28^.

Previous researches^29,30^ suggests a simple mid-frequency point calibration using a known resistor for the AD5933 chip, this method only works accurately for resistive elements like resistors. For components with capacitive or inductive properties (capacitors and inductors), their impedance varies across frequencies due to the imaginary component caused by capacitance and inductance. This is particularly relevant for piezoelectric transducers used in Structural Health Monitoring (SHM), which exhibit capacitive behavior alongside some resistance. The calibration resistor value (R_CAL_) should be equal to:

6 $$\:{R}_{CAL}=\frac{{Z}_{min}+{Z}_{max}}{3}$$

The minimal impedance $\:{Z}_{min}$ and maximal impedance $\:{Z}_{max}$ of the PZT element were determined through pre-tests. Based on these pre-test results, the calibration resistor $\:{R}_{CAL}$ was initially determined. Additionally, in the case of measuring pure resistance, the referential resistor $\:{R}_{FB}$ was chosen to be the same as the calibration resistor ($\:{R}_{FB}$ = $\:{R}_{CAL}$). Once $\:{R}_{CAL}$ and $\:{R}_{FB}$ values were established, they were connected to the AD5933 board at the desired position. The software dashboard allowed for configuring relevant board parameters. Subsequently, an impedance measurement could be conducted, and various parameters derived from real $\:{R}_{z}$ and imaginary $\:{I}_{z}$ data, such as magnitude $\:M$, gain factor $\:GF$, and phase $\:{\phi\:}_{AD5933}$, could be calculated. To determine the magnitude of known resistance R_CAL_ using real and imaginary impedance data from AD5933, the following equation was employed:

7 $$\:M=\sqrt{{R}_{Z}^{2}+{I}_{Z}^{2}}$$

Using the calculated magnitude $\:M$, the gain factor $\:GF$ could be obtained through the formula:

8 $$\:GF=\frac{{Y}_{CAL}}{M}=\frac{\frac{1}{{Z}_{CAL}}}{M}=\frac{\frac{1}{{R}_{CAL}}}{M}$$

Here, $\:{Y}_{CAL}$ and $\:{Z}_{CAL}$ represent the calibration admittance and impedance, respectively. It’s important to note that in this study, the calibrating resistor $\:{R}_{CAL}$ is used in place of impedance $\:{Z}_{CAL}$. Furthermore, the phase $\:{\phi\:}_{AD5933}$ could be computed using the general formula:

9 $$\:{\phi\:}_{Z}={{tan}}^{-1}(\frac{{I}_{Z}}{{R}_{Z}})$$

To acquire measurements of the free PZT patch, $\:{R}_{CAL}$ resistor was detached from the board and connected to the PZT patch. Impedance measurement using the AD5933 board was then performed, yielding real and imaginary data for impedance. In this case, the impedance magnitude of the free PZT patch could be determined with this equation:

10 $$\:{Z}_{PZT}=\frac{1}{GF.{M}_{PZT}}$$

Here, $\:{M}_{PZT}$ represents the impedance magnitude computed for the piezoelectric transducer using Eq. 7. Simultaneously, the phase $\:{\phi\:}_{sys}$ was calculated using Eq. 9. Using obtained phase values $\:{\phi\:}_{AD5933}$ and $\:{\phi\:}_{sys}$, the corrected phase $\:{\phi\:}_{PZT}$ for the PZT patch could be calculated as follows:

11 $$\:{\phi\:}_{PZT}\:=\:{\phi\:}_{SYS}\:-\:{\phi\:}_{AD5933}$$

Next, based on the corrected $\:{Z}_{PZT}$ (obtained in Eq. 10) and the phase $\:{\phi\:}_{PZT}$ (calculated in Eq. 11), the real $\:{R}_{PZT}$ and imaginary $\:{X}_{PZT}$ components of the PZT impedance can be calculated using the following equations:

12 $$\:{R}_{PZT}\:=\:\left|{Z}_{PZT}\right|\:*\:cos\left({\phi\:}_{PZT}\right)$$

13 $$\:{X}_{PZT}\:=\:\left|{Z}_{PZT}\right|\:*\:sin\left({\phi\:}_{PZT}\right)$$

Furthermore, the phase angle $\:{\theta\:}_{PZT}$ between the real and imaginary parts of the admittance can be obtained by taking the negative of $\:{\phi\:}_{PZT}$:

14 $$\:{\theta\:}_{PZT}\:=\:-{\phi\:}_{PZT}$$

Note that $\:{\theta\:}_{PZT}$ represents the phase angle between the real and imaginary parts of the admittance.

#### Impedance data acquisition

This section shows an in-depth calibrating and evaluation method applied to collect impedance data from sand specimens treated with FICP through the AD5933 evaluation board. Before taking impedance measurements, calibration of the AD5933 board was carried out as described in Section “EVAL -AD5933 chip and calibration”.

Step 1: A preliminary frequency sweep excitation test, spanning the frequency range of 30–100 kHz, was initially conducted using a commercial LCR meter. The obtained impedance measurements, having magnitude |$\:Z$| and phase $\:\theta\:$, helped the determination of calibration and reference resistors for the AD5933 system. These resistors, denoted as $\:{R}_{CAL}$ and $\:{R}_{FB}$, respectively, were given values of 10 kΩ (Fig. 3d). Furthermore, the frequency range of 30 to 100 kHz was found as the impedance-sensitive frequency range for the test specimen.

Step 2: The calibration resistor $\:{R}_{CAL}$ and the reference resistor $\:{R}_{FB}$ were subsequently integrated into their designated positions on the AD5933 board. The control software on the laptop was employed to pre-configure the measurement parameters of the AD5933 board. Utilizing these parameters, a sweep excitation with a frequency range of 30 to 100 kHz and a voltage amplitude of 1 V was administered to the calibration resistor. This method enabled the collection of the impedance response, containing magnitude and phase. After that analysis of these measurements allowed the estimation of the gain factor $\:GF$ and phase $\:{\phi\:}_{AD5933}$.

Step 3: After successful calibration, the calibration resistor $\:{R}_{CAL}$ was replaced with a PZT patch embedded in the sand. Using the AD5933 board with the exact predefined parameters that were used in the previous step, a frequency sweep excitation test was performed on the PZT patch. As a result, real and imaginary impedance values were obtained.

Step 4: The impedance measurements recorded in the step 3 underwent for calibration with the previously extracted calibration parameters: gain factor $\:GF$ and phase $\:{\phi\:}_{AD5933}$, as determined in step 2. This calibration procedure helped the measurement of the true impedance response of the test specimen.

### Mechanical and microstructural tests

#### Unconfined compression strength (UCS)

The Unconfined Compression Strength (UCS) test is an established laboratory technique used to measure the mechanical properties of MICP treated sand^31^. It offers an easy and fast method to determine the maximum axial stress that a cylindrical sample can withstand prior to a failure, in zero lateral confinement. In this research, the UCS test was applied to assess the influence of FICP treatment on the strength of sand specimens. The method of testing included applying a constant axial load at a controlled strain rate of 1 mm per minute to the treated sand specimens. Every specimen had a width of 38 mm and a height of 76 mm. Furthermore, the top and bottom surfaces of the cylinders were carefully smoothed to make sure uniformity and assure the load was imposed perpendicularly to the long axis of the specimen.

#### Calcium carbonate content (CCC) analysis

After the UCS test, the treated sand samples are crushed. The crushed samples are then dried in the oven for a period of two days. This removes any additional moisture inside the specimens, resulting in precise measurement of the dry weight. Measured 10-gram samples are then soaked in an acid solution (1 M hydrochloric acid, HCl) for 24 h. This results in dissolving the calcium carbonate precipitated due to FICP. Following the acid treatment, the specimens are completely rinsed to get rid of any remaining acid solution. They are then drained and dried again in an oven for 48 h. The mass difference between the initial dry mass ($\:{m}_{i}$) of the 10-gram sample before acid treatment and the final dry mass ($\:{m}_{f}$) after acid treatment and drying is calculated. The following formula was used to calculate the $\:{CaCO}_{3}$ content:

15 $$\:{CaCO}_{3}\:content\:\left(\%\right)=\frac{{m}_{i}-{m}_{f}}{{m}_{i}}\times\:100$$

#### SEM and EDX analysis

Scanning electron microscopy (SEM) and energy-dispersive X-ray spectroscopy (EDX) were employed to investigate the effects of FICP treatment on the microstructure and elemental composition of the sand samples. Following the treatment, the sand particles were meticulously separated and mounted onto SEM stubs using carbon tape. To mitigate electron beam charging during SEM analysis, the samples were sputter-coated with a thin layer of gold. A JEOL JSM-6510LV instrument operating at an accelerating voltage of 20 keV was used for the SEM analysis. The SEM images were captured at various magnifications to examine the microstructural alterations, particle morphology, and surface characteristics of the treated sand samples. The EDX analysis was carried out with an Oxford Instruments INCAx-act detector that was integrated with the same SEM equipment. The EDX detector recorded the energy and intensity of the X-rays emitted after providing the samples to an electron bombardment. Relevant data about the distribution as well as elemental composition of the treated sand samples were given by this analysis.

### Evaluation using statistical metrics

Evaluating EMI data for sand specimens treated with FICP typically involves comparing the measured EMI responses with a reference or baseline data. Statistical metrics commonly used for this purpose include Root Mean Square Deviation $\:RMSD$, Mean Absolute Percentage Deviation $\:MAPD$, and Correlation Coefficient $\:CC$^32^. These metrics can help quantify the accuracy and agreement between the treated and untreated EMI data. $\:RMSD$ measures the differences between the treated EMI data and the reference untreated data. It calculates the square root of the mean of the squared differences between corresponding data points. The formula for $\:RMSD$ is as follows:

16 $$\:RMSD=\sqrt{\frac{1}{n}{\sum\:}_{i=1}^{n}{({x}_{i}-{y}_{i})}^{2}}$$

$\:MAPD$ measures the percentage deviation between the treated EMI data and the reference data. It calculates the mean of the absolute percentage differences between corresponding data points. The formula for$\:\:MAPD$ is as follows:

17 $$\:MAPD=\frac{100}{n}{\sum\:}_{i=1}^{n}\left|\frac{{x}_{i}-{y}_{i}}{{y}_{i}}\right|$$

The correlation coefficient $\:CC$ measures the linear relationship between the treated and reference EMI data. It quantifies how well the two datasets vary together. The formula for the correlation coefficient is as follows:

18 $$\:CC=\frac{{\sum\:}_{i=1}^{n}({x}_{i}-\stackrel{-}{x})({y}_{i}-\stackrel{-}{y})}{\sqrt{{\sum\:}_{i=1}^{n}{({x}_{i}-\stackrel{-}{x})}^{2}}\sqrt{{\sum\:}_{i=1}^{n}{({y}_{i}-\stackrel{-}{y})}^{2}}}$$

Where, $\:n$ is the number of data points in the dataset, $\:{x}_{i}$ is the EMI measurement for the treated specimen at index $\:i$, $\:{y}_{i}$ is the EMI measurement for the reference specimen at index $\:i$, $\:\stackrel{-}{x}$ and $\:\stackrel{-}{y}$ are the means of the treated and reference EMI data, respectively.

A lower $\:RMSD$ and $\:MAPD$ value indicates a better agreement between the treated and reference EMI data. The correlation coefficient ranges between − 1 and 1. A value close to 1 indicates a strong positive linear correlation, while a value close to -1 indicates a strong negative linear correlation. A value close to 0 indicates a weak or no linear correlation. By calculating these statistical metrics, the effectiveness of the FICP treatment on sand specimens can be assessed based on the EMI data and compare it to the reference or baseline data.

## Results and discussion

### Impedance changes during FICP treatment

**Fig. 4:**
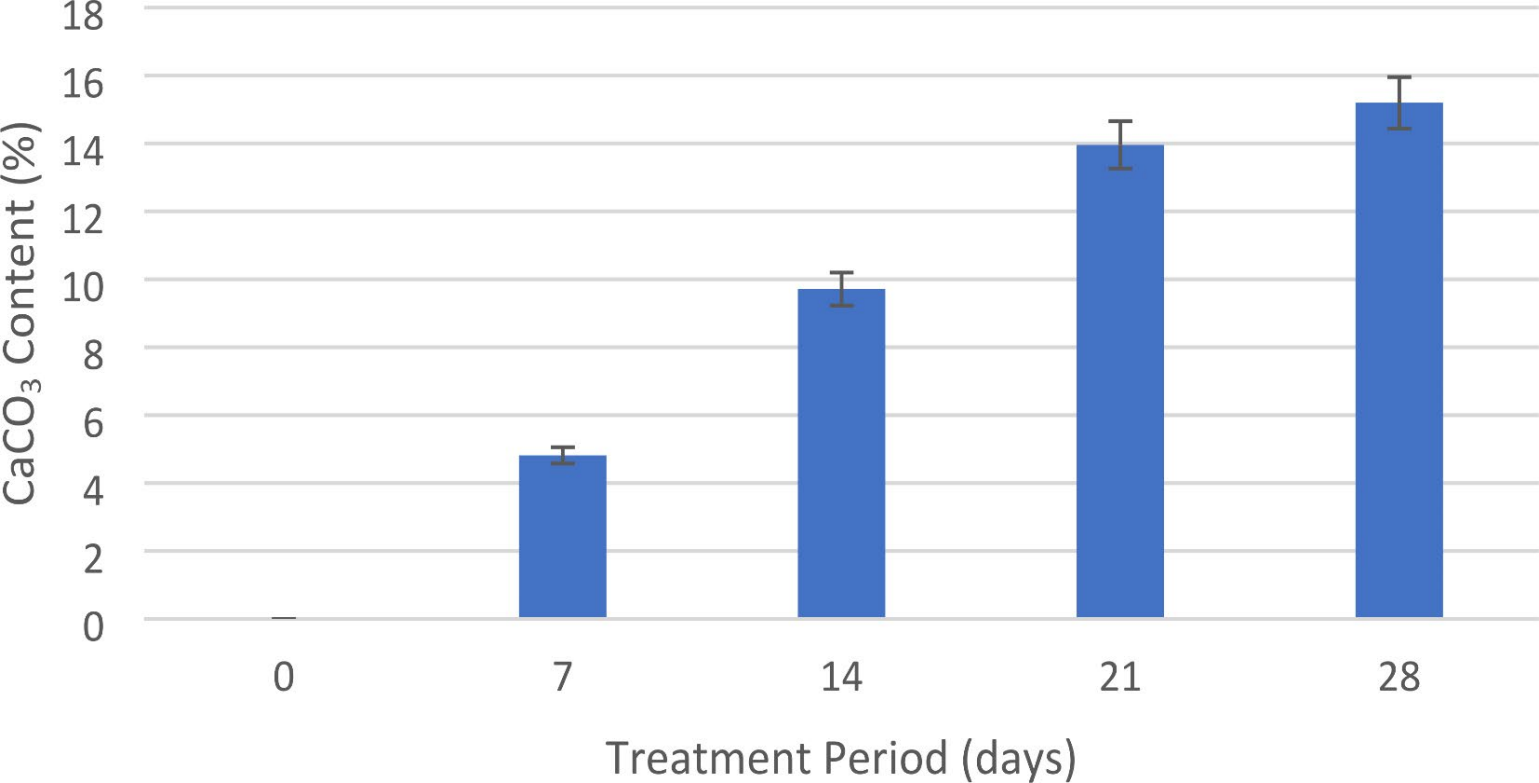
Change in CaCO_3_ content (%) with FICP treatment time.

**Fig. 5:**
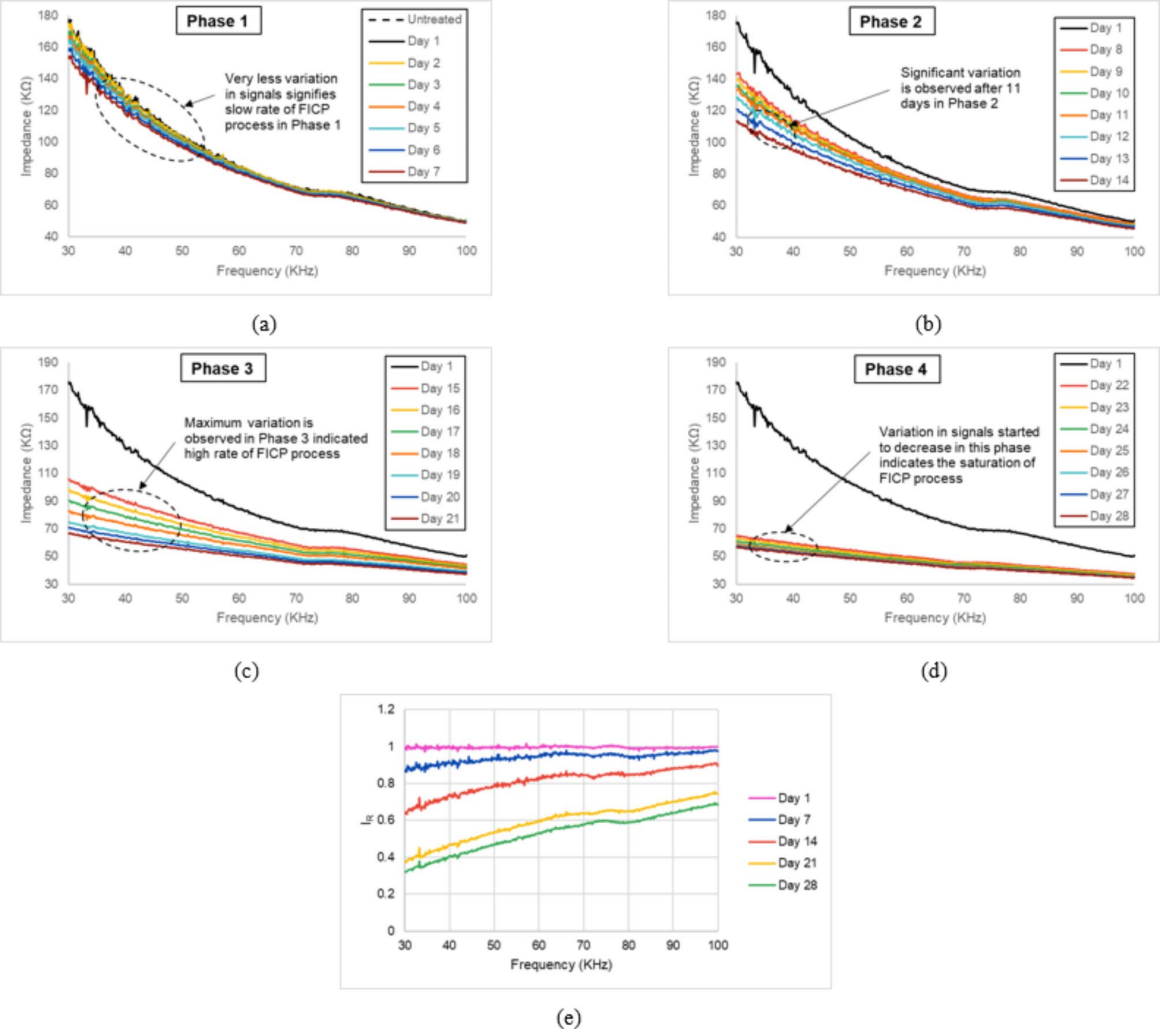
Impedance signals variation from day 1 to day 28 in the frequency range of 30–100 kHz, (a) Day 1–7 (Phase 1), (b) Day 8–14 (Phase 2), (c) Day 15–21 (Phase 3), (d) Day 22–28 (Phase 4), and (e) Temporal variation of I_R_ (ratio of treated impedance at the end of each phase and untreated impedance).

The FICP treatment process was studied over a 28-day period of time, separated into four phases: Day 1–7 (Phase 1), Day 8–14 (Phase 2), Day 15–21 (Phase 3), and Day 22–28 (Phase 4). At the completion of each phase, CCC analysis was carried out (Fig. 4). The phase 1 of the experiment, comprising days 1 to 7 of the FICP treatment on untreated sand, exhibited a very little shift in the EMI signals (Fig. 5a). This small variation suggests low precipitation of CCC inside the sand matrix during this period. This gives with the knowledge that the initial stages of FICP treatment are defined by low fungal activity and low calcium carbonate precipitation. The CCC analysis at the completion of this phase validates this by giving a value of only 4.81%. Phase 2, encompassing days 8 to 14 of the FICP treatment, observed a considerable shift compared to the initial phase (Fig. 5b). A considerable shift in the EMI curves became obvious, showing a definite acceleration in the precipitation of $\:{CaCO}_{3}$ inside the sand matrix. This significant shift shows that fungal activity had gained outstanding rise throughout this period. The curves clearly revealed a greater gap compared to Phase 1, providing a significant visual indicator for the ongoing changes to the mechanical properties of the treated sand. This expanding gap can be defined to the enhancing calcium carbonate deposits within the sand particles’ gaps. Phase 2 implies a more active stage where the biological mechanisms that trigger calcium carbonate precipitation function at a considerably higher rate. This enhanced activity has been confirmed by the CCC determined at the end of this phase. The measured figure of 9.71% represents a near doubling of the calcium carbonate level compared to Phase 1 (4.81%). This considerable rise gives quantifiable proof for the higher precipitation activity during this period. Phase 3 (15 to 21 days), noticed a continuation and strengthening of the patterns observed in Phase 2 (Fig. 5c). The EMI curves revealed considerably more notable maximum shifts compared to the prior phases. This alteration suggests a continuing and rapid precipitation of $\:{CaCO}_{3}$ inside the sand matrix. This broader gap reflects more significant modifications to the mechanical characteristics of the treated sand. As the calcium carbonate continues to precipitate within the spaces between sand particles, it works as a binding agent, affecting the overall rigidity and stiffness of the sand. This greater rigidity correlates to a wider spacing in the EMI signals. Finally, in Phase 4, Day 22–28, the EMI curves appeared to be quite close together, signifying approaching finishing of the FICP treatment process (Fig. 5d). This observation matches exactly with the expected consequence of a successful FICP treatment. Figure 5(e) presents the I_R_, defined as the ratio of the impedance of the treated sample to the untreated sample, measured at the start and end of each phase: Day 1, Day 7, Day 14, Day 21, and Day 28. The I_R_ values provide insight into the temporal changes in the sand’s electromechanical behaviour due to the applied FICP treatment over time. On Day 1, the I_R_ remains close to 1, indicating minimal difference between the treated and untreated samples, as the treatment has not yet significantly influenced the impedance properties. The progressive decrease in I_R_ over time reflects the impact of the FICP treatment time, with the most significant changes occurring between Days 7 and 21. The stabilization after Day 21 suggests that the FICP treatment’s effects have fully manifested, and the electromechanical properties of the sample are no longer evolving at the same rate. The method is meant to achieve a specified level of strengthening and stabilization inside the treated sand. Once this aim is accomplished, the biological activity that causes precipitation naturally decreases down as the entire system approaches a state of equilibrium. The study successfully proves EMI as a viable technique for real-time monitoring of FICP. The correlation between impedance changes and CCC shows the technique can be utilized to enhance treatment processes. Calcium carbonate precipitation forms strong, localized connections between sand particles^33^. These connections strengthen resistance to shear stresses, a significant determinant in sand strength. The higher the amount of calcium carbonate crystals generated, the stronger the inter-particle bonding and enhanced strength^34^. Factors including microbial activity, nutrition availability, and response time determine the amount of precipitation. The size, shape, and surface roughness of sand particles additionally impact the nucleation and adherence of calcium carbonate crystals^33,35^.

### Effect of treatment time on unconfined compressive strength (UCS)

**Fig. 6:**
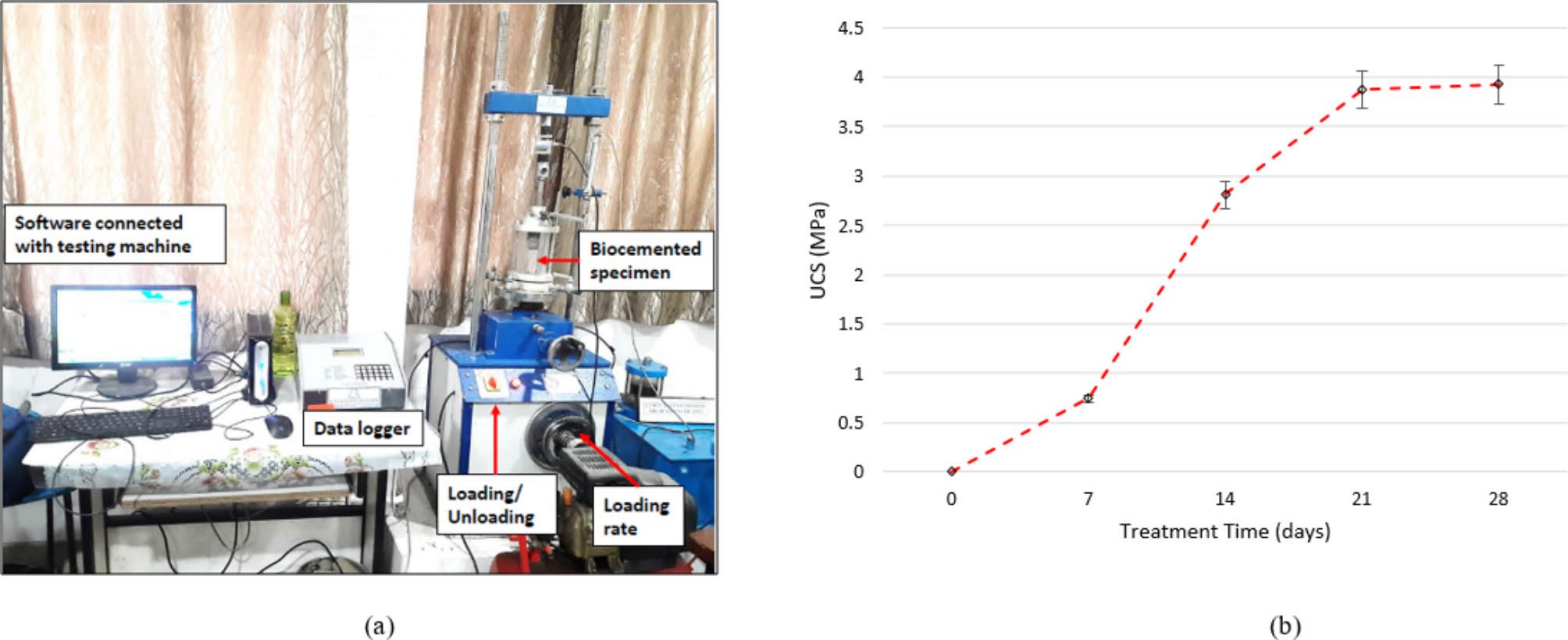
(a) Experimental setup for UCS tests, (b) Variation of UCS with treatment time.

This section evaluates the crucial part that treatment period has in improving the UCS of FICP treated sand samples. The experiment studied the effect of the duration of treatment by *A. niger* fungus for four different periods: 7 days, 14 days, 21 days, and 28 days. The UCS was assessed for each treatment period of time, with outcomes provided as average values. The results, as displayed in Fig. 6, reveal an interesting trend—a gradual rise in UCS with duration of treatment. This suggests that the fungal treatment has a favourable and time-dependent influence on the UCS of the sand samples. Even during the first week of treatment, the fungus displayed a rapid influence on the mechanical properties of the sand. This is obvious from the increased UCS values reported after just 7 days. The average UCS after this duration was observed at 0.745 MPa, with a standard deviation of 0.0128. This represents an improvement compared to the UCS of untreated sand. Increasing the treatment time to 14 days resulted in a substantial rise in the average UCS. The number achieved was a remarkable 2.81 MPa, with a low standard deviation of 0.046. This significant enhancement highlights the increasing extent of the fungal mycelium’s effect on the sand’s structural integrity during a 2-week period of time. Further treatment advancement, up to 21 days, provided an average UCS of 3.87 MPa, with a standard deviation of 0.077. This continuous trend shows that the A. niger fungus exerted an ongoing strengthening impact on the sand samples. However, findings also indicate at a possible plateau in the strengthening impact. The average UCS achieved a saturation point at 28 days, with a value of 3.93 MPa (standard deviation 0.046). This shows that even though the treatment remains to be useful beyond 21 days, the degree of UCS improvements could decline after this stage.

Previous Studies have consistently showed a positive relationship between MICP treatment period and UCS^36–39^. As treatment time increases, the amount of calcium carbonate precipitation inside the sand matrix likewise increases. This additional cementing agent bonds the sand particles together, giving to a considerable improvement of compressive strength. Wen^39^ shows UCS values increased proportionally with the amount of MICP treatment cycles. The selected MICP technique (submersion or percolation) may influence in terms of treatment duration. Percolation procedures likely to be more effective, reaching greater UCS in shorter times in comparison to submersion^39^. Particle size, morphology, and initial sand properties may also influence the relation among treatment time and UCS. For example, studies conducted by Zeitouny^38^ indicates angular sands can have a greater UCS increase with a shorter treatment period than spherical sands. Although longer treatment periods usually contribute to higher UCS, there is an optimization aspect to keep in mind. After a certain period of time, the rate of UCS increase could reduce as it started after 21 days in our experiment. Furthermore, longer treatment times lead to higher project expenses. Therefore, determining the best treatment duration for a certain application is essential.

### Variation of statistical metrics for different treatment periods

**Fig. 7:**
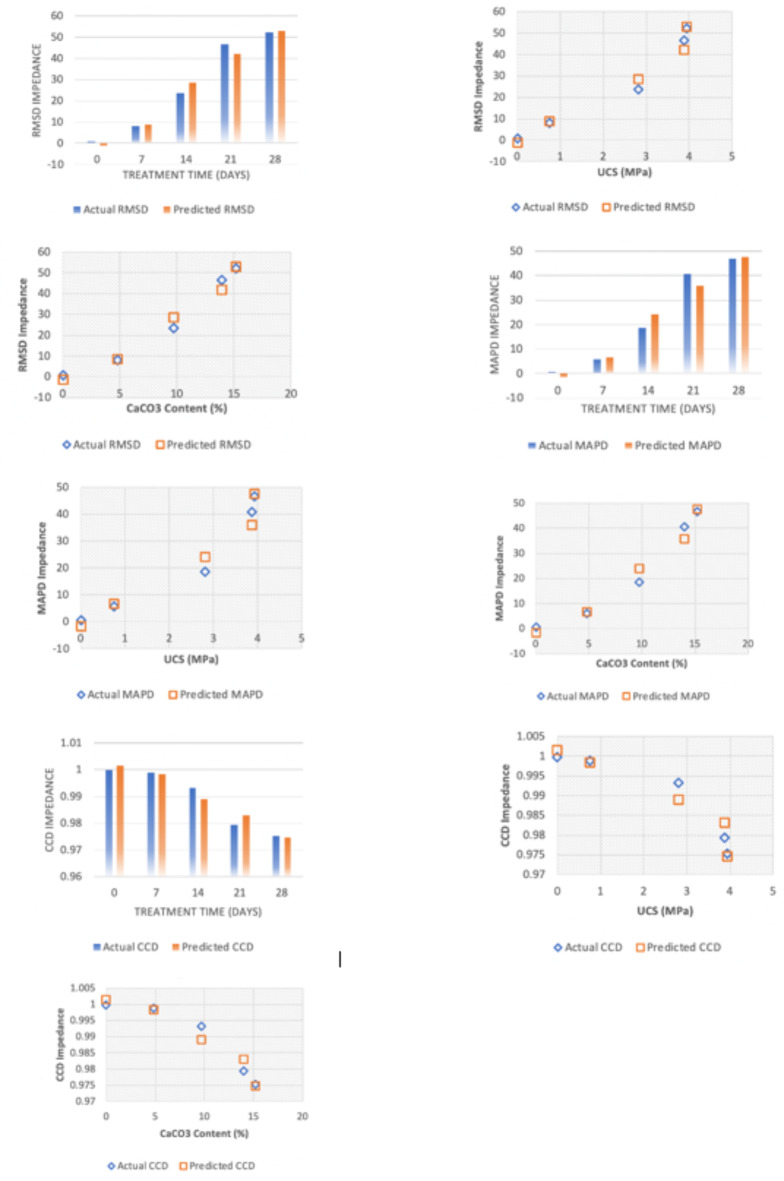
Model fitting and evaluation process for the impedance measures (RMSD, MAPD, and CCD).

This section evaluated the impact of FICP treatment its duration, which stem UCS, and CCC on the impedance of sand samples by 3 statistical metrics such as RMSD, MAPD, and CCD. The findings indicated a clear pattern of increased RMSD and MAPD impedance metrices with increasing duration of treatment (Fig. 7). This implies that the FICP treatment successfully influence the physical properties of the sand, resulting to significantly higher impedance RMSD and MAPD metrices. The RMSD values grew consistently from 0.7279 after a treatment time of 1 day to 52.2821 for 28 days. This suggests an increasing separation from the baseline impedance while the treatment continued. Likewise, RMSD, MAPD values too indicated an increase with treatment time, varying from 0.5745% for 1 day to 46.7718% for 28 days. This shows a progressively bigger absolute difference in percentage terms among the untreated and the treated samples when treatment duration increased. The CCD values were continuously high during the course of the experiment, varying between 0.99 (nearly perfect agreement) for 1–14 days to 0.97 for 21–28 days. This reveals a significant correlation among the impedance values of the untreated and the FICP-treated sand samples with the increase of treatment time. Between the three metrics, RMSD displays the most reliable and apparent pattern during the treatment period, which makes it the best functioning indicator when it comes of representing the treatment’s impact on impedance. The results of this investigation reveal that FICP treatment effectively changes the impedance properties of sand, and RMSD acting as a trustworthy indicator of these alterations. The outcomes from this investigation reveal that FICP treatment successfully affects the impedance properties of sand. Multiple linear regression equations for the RMSD, MAPD and CCD values of impedance along with treatment time, UCS, and CaCO_3_ content are developed. The equations relating to RMSD_impedance_, MAPD_impedance_, CCD_impedance_, treatment time (X), UCS (Y), calcium carbonate content (Z) are as follows:

19 $$\:{RMSD}_{impedance}={a}_{1}{X}^{{a}_{2}}{Y}^{{a}_{3}}{Z}^{{a}_{4}}$$

20 $$\:{MAPD}_{impedance}={b}_{1}{X}^{{b}_{2}}{Y}^{{b}_{3}}{Z}^{{b}_{4}}$$

21 $$\:{CCD}_{impedance}={c}_{1}{X}^{{c}_{2}}{Y}^{{c}_{3}}{Z}^{{c}_{4}}$$

A statistical technique known as multiple regression analysis was employed to estimate the coefficients (a_1_ – a_4_), (b_1_ – b_4_), and (c_1_ – c_4_). This involved transforming the original equations into a linear format by applying a natural logarithm to both sides. Subsequently, the analysis yielded the coefficient values that provide the optimal fit for the available data. The resulting equations are:

22 $$\:{RMSD}_{impedance}=-1.22\:{X}^{1.81}\:{Y}^{7.82}{\:Z}^{-1.79}$$

23 $$\:{MAPD}_{impedance}=-1.52{\:X}^{2.01}{\:Y}^{7.21}\:{Z}^{-2.34}$$

24 $$\:{CCD}_{impedance}=1.001\:{X}^{-0.0016}\:{Y}^{-0.005}\:{Z}^{0.0024}$$

Multiple linear regression analysis was undertaken to build models which estimate these impedance measurements based on the contributing parameters (treatment duration, UCS, and CCC). The high R-squared values (0.975 for RMSD, 0.965 for MAPD, and 0.934 for CCD) show an excellent fit among the models and the data (Fig. 7). This suggests the models effectively represent the correlation between the independent variables (treatment time, UCS, and CCC) and related impedance values.

### SEM and EDX analysis

**Fig. 8:**
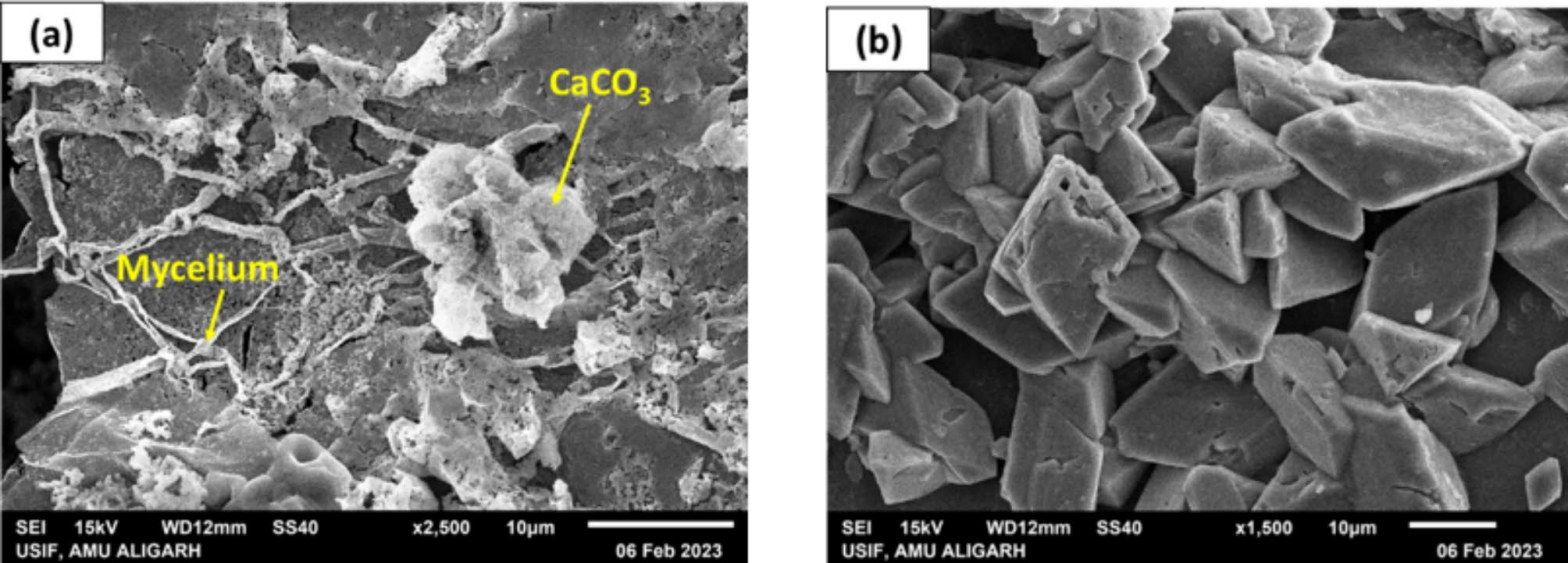
SEM images of FICP treated Sand after 28 days.

Scanning Electron Microscopy (SEM) analysis was carried out on the FICP-treated sand specimens to investigate the morphology and distribution of the precipitated calcium carbonate on the microscopic level. SEM examination indicated the extensive spread of the fungal hyphae in sand matrix. Figure 8a display a highly dense and complex web of filaments widely spreading and branching over the sand particles. This web-like network denotes the successful growth of the fungus in the sand matrix, an essential phase for the successful FICP treatment. In addition, SEM pictures offered an understanding of the significance of the fungal web in promoting $\:{CaCO}_{3}$ precipitation after 28 days treatment. The immense surface area attached by the complicated mycelial network (Fig. 8a) acted as numerous nucleation sites enabling the precipitation of $\:{CaCO}_{3}$ crystals. Figure 8b shows the morphology of the noticed crystalline mineral deposits, displaying typical rhombohedral forms. This specific morphology is a widely recognized property of calcite, which is the most thermodynamically stable form of calcium carbonate^38^. The finding validates the successful production of calcite crystals using the FICP method.

**Fig. 9:**
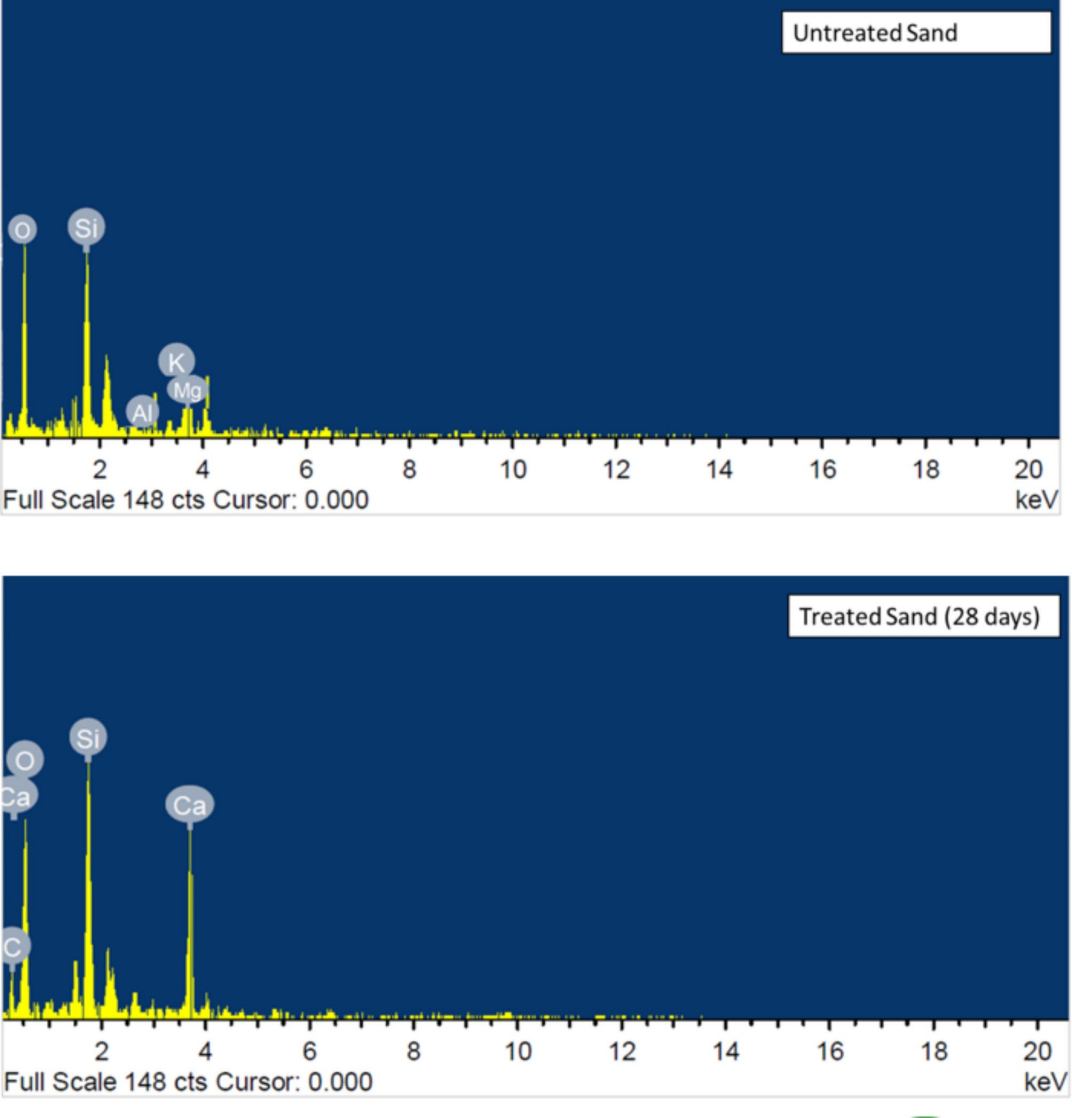
EDX analysis of untreated and FICP treated sand.

Energy-Dispersive X-ray Spectroscopy (EDX) analysis effectively assessed the influence of FICP treatment on the elemental composition of the sand (Fig. 9). This approach found several elements present, giving information about $\:{CaCO}_{3}$ production and its interaction on sand grains. The untreated sand largely comprised silicon ($\:Si$) and oxygen ($\:O$), which is normal for sand having silicon dioxide ($\:{SiO}_{2}$). Small quantities of aluminum ($\:Al$), potassium ($\:K$), and magnesium ($\:Mg$) have been identified. Oxygen, being the most prevalent element, probably originates from the sand matrix ($\:{SiO}_{2}$). The existence of calcium ($\:Ca$) in the treated samples acted as the signal of $\:{CaCO}_{3}$ production. Compared with the untreated sample, the level of calcium was considerably greater in samples treated for 28 days. Carbon ($\:C$), a further important part of $\:{CaCO}_{3}$, was discovered in the treated samples, confirming the effective precipitation of calcite crystals. The carbon content also revealed a substantial increase comparing with the untreated sand. These results indicate that FICP successfully alters the surface composition of the sand particles. The presence of $\:{CaCO}_{3}$ precipitates likely increases interparticle bonding, perhaps resulting in enhanced mechanical properties such UCS^8,38^.

## Conclusions

This study has proven the effectiveness of FICP in enhancing the mechanical properties of sand. The study specifically highlights the use of EMI for real-time monitoring. During a 28-day treatment period, the impedance changes recorded using PZT based EMI offered significant insights on the development and effectiveness of the FICP process. This demonstrated the potential of FICP for field application in soil stabilisation. The key findings of this research are as follows:

i. During Phase 1, which lasted for 7 days, there were very slight changes in the EMI signals, suggesting that there was limited fungal activity. Additionally, there was an early formation of calcium carbonate, with a CCC value of 4.81%. During Phase 2, which lasted from Days 8 to 14, there were noticeable changes in the EMI curves that indicated a faster growth of fungi and a significant increase in CCC (9.71%). During Phase 3 (Days 15–21), there were persistent and significant increases in impedance, indicating the continuous and rapid formation of CaCO3 (calcium carbonate) deposits. This process resulted in increased stiffness and rigidity of the sand. During Phase 4 (Days 22–28), the stabilisation of EMI curves indicated that the FICP treatment was nearing completion, suggesting a successful stabilisation process.
ii. The UCS tests demonstrated a direct correlation between the period of treatment and the increase in strength over time. UCS values increased from 0.745 MPa at 7 days to 3.87 MPa at 21 days, with a minor plateau reported at 28 days (3.93 MPa). This pattern underlines the ongoing strengthening given by the FICP process, with the fungal mycelium making an important contribution to the overall strength of the sand.
iii. The RMSD, MAPD, and CCD measures exhibited strong trends related with treatment time, UCS, and CCC. RMSD identified as the most reliable metric, with values increasing continuously across the treatment period. Multiple linear regression (MLR) analysis yielded robust models relating impedance variations in treatment duration, UCS, and CCC, with high R-squared values (0.975 for RMSD, 0.965 for MAPD, and 0.934 for CCD).
iv. The images from the SEM indicated substantial fungal hyphae spread and calcite crystal production, proving the success of FICP to induce calcium carbonate precipitation. EDX analysis indicated considerable increases in calcium and carbon content in treated samples, suggesting effective calcite deposition and increased interparticle bonding.

## Limitations and future scope

Although the positive outcomes of this investigation, numerous limitations need to be addressed. First, the introduction of *Aspergillus Niger* fungus, however innovative, establishes uncertainty in the FICP process due to the biological nature of the microbe. Factors such as nutrient availability, pH levels, and ambient conditions can considerably affect the effectiveness and uniformity of calcium carbonate precipitation. Future research might gain insight from examining a broader spectrum of fungus or genetically modified strains to enhance the robustness and dependability of the FICP process. An additional limitation is in the frequency range of the AD5933 impedance chip, that is limited to maximum 100 kHz. Although this range was enough to identify significant EMI signal shifts related to calcium carbonate production, increasing the frequency range might offer more thorough insights regarding the microstructural changes inside the treated sand. Furthermore, the calibration method, while extensive, relies on the accuracy of the commercial LCR meter. Differences in calibration standards or apparatus could generate disparities in the EMI readings. The experimental tests concentrated on certain treatment periods (7, 14, 21, and 28 days), giving an overview of the FICP process at these periods of time. Yet, the constantly changing character of microbial activity and calcium carbonate precipitation indicates that more continuous monitoring might provide a deeper knowledge of the time history of soil strengthening. Integrating more frequently observations or using real-time data collecting technologies could enhance the level of detail of the study. In this investigation, established assessment criteria such as CCD, RMSD, and MAPD were used to evaluate the EMI signal changes. While the results give important statistical understandings, but don’t correspond to the physical nature of the monitored structure, such as the FICP treated sand. Furthermore, these steps are extremely based on the choice of the frequency band, which could alter the accuracy of the analysis. Recent studies have focused on applying deep learning to automatically interpret EMI signatures, enabling a more comprehensive approach to understanding the underlying physical processes. In future advanced deep learning algorithms may be referred to examine these limits and prospective breakthroughs.

Looking forward, there are various areas for future research. One potential possibility is the combination of powerful machine learning algorithms with EMI spectroscopy data to generate prediction models for soil strength development. This should enable more accurate and automatic monitoring of MICP-treated sand, easing large-scale adoption in field applications. Additionally, increasing the scope of the study to include diverse soil types and external factors would provide a more comprehensive knowledge of the usefulness and limitations of the FICP technique. Further studies should also investigate the long-term durability and environmental impact of MICP-treated sand. While the immediate strength gains are noticeable, understanding the endurance and stability of the calcium carbonate connections under diverse environmental pressures is vital for practical applications. Studies on the biodegradability of the precipitated calcium carbonate and its interaction with soil and groundwater chemistry could provide useful insights into the sustainability of this soil treatment technology. The development of more complex and user-friendly EMI monitoring devices could promote widespread usage of this technology in geotechnical engineering. Collaborations with industry partners to produce portable, cost-effective, and resilient EMI devices could bridge the gap between laboratory research and field deployment, making real-time monitoring of soil strength development a regular practice in construction and infrastructure projects. The potential societal advantages of this research are enormous. MICP-treated sand offers an environmentally acceptable alternative to typical soil stabilization technologies, which sometimes rely on chemical additions that can be damaging to the environment. By improving the strength and durability of soils utilizing naturally occurring processes, this technology can contribute to more sustainable construction techniques. The real-time monitoring capabilities given by EMI spectroscopy further ensure the effectiveness and safety of soil treatments, possibly reducing the probability of structural failures and associated expenses.

## Data Availability

Data is available on reasonable request made to author Jamal Ahmad.
